# Prevalence and determinants of cesarean section delivery among Somali women: a multivariable analysis of a nationally representative survey

**DOI:** 10.1080/16549716.2026.2747464

**Published:** 2026-10-14

**Authors:** Fatima Mohamud Ahmed

**Affiliations:** Faculty of Medicine and Health Science, Jamhuriya University of Science and Technology, Mogadishu, Somalia

**Keywords:** Maternal health, emergency obstetric care, health service accessibility, maternal mortality, health inequities

## Abstract

**Background:**

Cesarean section is a life-saving obstetric intervention when medically indicated, yet access remains severely limited in Somalia, where maternal morbidity and mortality are high.

**Objective(s):**

To determine the magnitude of cesarean section delivery and identify factors associated with its utilization among Somali women using nationally representative survey data.

**Methods:**

A secondary analysis of the 2020 Somali Health and Demographic Survey was conducted using data from 23,445 women aged 15–49 years. Odds ratios (ORs) with 95% confidence intervals (CIs) were reported, with statistical significance set at *p* < 0.05.

**Results:**

The prevalence of cesarean section was 0.3% (61/23,445). Compared with women aged ≥ 35 years, those aged 25–34 years (OR = 6.483; 95% CI: 2.160–19.458; *p* = 0.001) and <25 years (OR = 6.293; 95% CI: 2.422–16.351; *p* < 0.001) had statistically significantly higher odds of cesarean delivery. Rural residence (OR = 0.177; 95% CI: 0.091–0.346; *p* < 0.001) and low household wealth (OR = 0.243; 95% CI: 0.097–0.611; *p* = 0.003) were statistically significantly associated with lower odds of cesarean delivery. Women with ≥6 children had statistically significantly higher odds of cesarean delivery (OR = 3.960; 95% CI: 2.072–7.569; *p* < 0.001). Breastfeeding was not significantly associated with cesarean delivery (*p* = 0.079).

**Conclusions:**

Cesarean section utilization in Somalia remains extremely low, indicating substantial inequities in access to emergency obstetric care. Maternal age, parity, place of residence, and household wealth were associated with cesarean delivery.

## Paper Context

- **Main findings:** Cesarean section delivery remains exceptionally rare among Somali women, with significant disparities associated with maternal age, household wealth, parity, and place of residence, indicating substantial inequities in access to emergency obstetric care.
- **Added knowledge:** This study provides the first nationally representative evidence on the prevalence and determinants of cesarean section delivery in Somalia, filling an important evidence gap for conflict-affected and resource-limited settings.
- **Global health impact for policy and action:** Expanding equitable access to comprehensive emergency obstetric services, strengthening referral systems, reducing financial and geographic barriers, and promoting person-centered family planning can help improve maternal and newborn outcomes in Somalia and similar low-resource settings.

## Background

Cesarean section is a life-saving obstetric procedure when medically indicated, yet its use varies widely across countries and population groups [1]. According to the World Health Organization, the rate of cesarean section deliveries should range between 10% and 15% at the population level [2]. This range is considered optimal as it strikes a balance between the life-saving benefits of Cesarean section and the risks of unnecessary surgical intervention [2]. Global cesarean section utilization has risen sharply over the past two decades, increasing from approximately 7% in 1990 to 21% today [1,3]. Projections suggest that this trend may continue, with cesarean section rates potentially reaching 29% by 2030 [3]. This upward trend is not uniform across populations and reflects substantial socioeconomic, health-system, and cultural disparities [3]. While high-income countries often face concerns of overuse [4,5], many low-income settings continue to struggle with inadequate access to emergency obstetric care [6].

A range of interacting factors influences the utilization of cesarean sections across populations. Socioeconomic position, including household wealth and maternal education, often determines access to skilled obstetric services, with wealthier and more educated women more likely to deliver in facilities capable of performing cesarean section [7–9].

Demographic factors such as maternal age, parity, and place of residence influence cesarean section use, with older, first-time mothers and urban residents typically having higher rates due to increased risk and better access to services [7,10]. Cultural beliefs and community attitudes toward childbirth also influence care-seeking behavior. At the same time, health-system factors – including staff availability, referral systems, facility capacity, and provider decisions – ultimately determine whether women can receive timely, life-saving cesarean section when complications occur [11,12].

In Somalia, maternal and neonatal health indicators remain among the poorest in the world, influenced by decades of conflict, fragile health systems, limited skilled birth attendance, and stark disparities between urban and rural areas [13]. Approximately 80% of maternal deaths are preventable through timely access to quality obstetric care provided by skilled health professionals, including emergency obstetric and newborn care [14,15].

Emerging evidence suggests that socioeconomic status, education, place of residence, and cultural beliefs strongly influence maternal health-seeking behavior in Somalia [16–18]; however, nationally representative evidence on the determinants of cesarean section delivery remains lacking. Although numerous studies have examined factors associated with cesarean section utilization in other low- and middle-income countries, their findings cannot be directly generalized to Somalia because of its unique context, characterized by prolonged conflict, a fragile health system, nomadic populations, limited access to emergency obstetric care, and one of the world’s lowest reported cesarean section rates.

The objective of this study was to determine the prevalence of cesarean section delivery and identify its associated socioeconomic, demographic, and reproductive determinants among Somali women using nationally representative data from the 2020 Somali Health and Demographic Survey (SHDS). We hypothesized that maternal age, socioeconomic status, place of residence, and parity would be independently associated with cesarean section delivery. By providing the first nationally representative analysis of cesarean section determinants in Somalia, this study addresses an important evidence gap and has the potential to generate context-specific evidence that may inform maternal health policies, guide resource allocation, strengthen emergency obstetric care and referral systems, and support efforts to improve equitable access to life-saving cesarean section services in Somalia and other conflict-affected and low-resource settings.

## Methods

### Study setting

This study was conducted in Somalia, a country in the Horn of Africa characterized by persistent conflict, fragile health infrastructure, and sociocultural norms that influence reproductive health and healthcare-seeking behavior. These challenges, together with widespread poverty and limited access to formal education and healthcare services, create a complex environment for reproductive health interventions.

### Data source and study design

This study was a secondary analysis of the 2020 SHDS, conducted by the Somali National Bureau of Statistics (SNBS). The survey collected data from 18 regions using a stratified sampling design comprising urban, rural, and nomadic populations. Banadir region was classified as entirely urban because it consists predominantly of Mogadishu, the capital city, and does not include separate rural or nomadic strata in the survey sampling framework. Because of field security challenges that limited safe access for data collectors, the three strata of Lower Shabelle and Middle Juba, as well as the rural and nomadic strata of Bay region, were excluded from the survey. The SHDS dataset and supporting documentation were obtained from the Somali National Data Archive (SoNADA) microdata portal (https://microdata.nbs.gov.so/index.php/catalog/50/get-microdata).

### Study population and eligibility criteria

Women aged 15–49 years were eligible for inclusion if they had complete information on their childbirth history, including the mode of delivery for their most recent birth, and complete data for all explanatory variables included in the regression analysis. Records with missing information on the outcome or explanatory variables were excluded. After applying these criteria, a total of 23,445 women were included in the final analysis.

### Variables

The outcome variable was cesarean section, defined according to the SHDS as surgical delivery reported by the mother for her most recent birth. Vaginal delivery served as the reference category.

Independent variables were selected based on evidence from previous epidemiological studies on determinants of cesarean section delivery, their theoretical relevance, and their availability in the survey dataset. The variables included maternal age, place of residence, region of residence, educational level, household wealth index, parity, current breastfeeding status, preceding birth interval, and child survival status.

Maternal age was categorized as <25 years, 25–34 years, and ≥35 years (reference category). Place of residence was categorized as urban (reference category) and rural. Region of residence was based on the administrative regions included in the survey. Educational level was categorized as no formal education, primary, secondary, and higher education, with higher education as the reference category.

Household wealth index was categorized as low, middle, and high-income, with high-income as the reference category. The wealth index was derived using principal component analysis according to the standard methodology used in demographic and health surveys [19].

Parity was categorized as ≤5 children (reference category) and ≥6 children. Current breastfeeding status was categorized as no (reference category) and yes. Preceding birth interval was defined as the interval between the woman’s two most recent live births and categorized as <24 months and ≥24 months, with ≥24 months as the reference category [20,21]. Child survival status was defined according to whether the woman’s most recent child was alive or deceased at the time of the survey interview, with alive as the reference category.

### Data analysis

Data analysis was conducted in three stages. First, descriptive statistics were used to summarize the sociodemographic and obstetric characteristics of the study participants, with categorical variables presented as frequencies and percentages. Second, bivariate logistic regression analyses were performed to assess the association between each candidate explanatory variable and cesarean section delivery. Crude odds ratios (CORs) with corresponding 95% confidence intervals (CIs) were calculated. Variables that demonstrated statistical significance in the bivariate analyses were subsequently entered into the final multivariable logistic regression model to identify factors independently associated with cesarean section delivery. Adjusted odds ratios (AORs) with corresponding 95% CIs were reported, and statistical significance was determined at *p* < 0.05.

Multicollinearity among the predictor variables included in the multivariable model was assessed using variance inflation factors (VIFs). A VIF value below 5.0 was considered to indicate no problematic multicollinearity. In the present analysis, VIF values ranged from 1.001 to 1.303, indicating no evidence of problematic multicollinearity.

## Results

We analyzed data from 23,445 women aged 15–49 years who met the study inclusion criteria from the SHDS 2020. Among them, 1,746 (7.4%) were younger than 25 years, 9,754 (41.6%) were aged 25–34 years, and the majority, 11,945 (50.9%), were 35 years or older. Regarding place of residence, 10,784 (46.0%) lived in urban areas, while 12,661 (54.0%) resided in rural settings. By regional distribution, the highest proportions of respondents were from Banadir (2,408; 10.3%), Sool (2,360; 10.1%), and Togdheer (1,959; 8.4%), whereas Bay (513; 2.2%) had the smallest representation. Most women in the study, 20,388 (87.0%), had never attended formal school. More than half were from high-income households (13,963; 59.6%), while smaller numbers came from middle- (4,792; 20.4%) and low-income (4,690; 20.0%) families.

The survival status of children indicated that the majority, 22,691 (96.8%), were alive, while 754 (3.2%) were reported deceased. Birth intervals were nearly evenly distributed, with 11,599 (49.5%) having intervals shorter than 24 months and 11,846 (50.5%) having intervals of 24 months or longer. Regarding parity, 14,811 (63.2%) of women had six or more children, while 8,634 (36.8%) had five or fewer. Delivery by cesarean section was extremely rare, reported in only 61 (0.3%) cases, compared with 23,384 (99.7%) who delivered vaginally. A total of 8,898 (38.0%) women were currently breastfeeding at the time of the survey, whereas 14,547 (62.0%) were not (Table 1).

**Table 1.** Descriptive characteristics of the study population (*n* = 23,445).

Variable	Category	Frequency (Percent)
Age (in 5-year groups)	<25 years	1,746 (7.4%)
	25–34 years	9,754 (41.6%)
	≥35 years	11,945 (50.9%)
Type of place of residence	Urban	10,784 (46.0%)
	Rural	12,661 (54.0%)
Region	Awdal	1,293 (5.5%)
	Woqooyi Galbeed	1,895 (8.1%)
	Togdheer	1,959 (8.4%)
	Sool	2,360 (10.1%)
	Sanaag	2,150 (9.2%)
	Bari	1,240 (5.3%)
	Nugaal	1,322 (5.6%)
	Mudug	1,322 (5.6%)
	Galgaduud	1,078 (4.6%)
	Hiraan	1,159 (4.9%)
	Middle Shabelle	1,150 (4.9%)
	Banadir	2,408 (10.3%)
	Bay	513 (2.2%)
	Bakool	1,414 (6.0%)
	Gedo	986 (4.2%)
	Lower Juba	1,196 (5.1%)
Highest educational level	No education	20,388 (87.0%)
	Primary	2,129 (9.1%)
	Secondary	711 (3.0%)
	Higher	217 (0.9%)
Sex of child	Male	12,342 (52.6%)
	Female	11,103 (47.4%)
Wealth index	Low	4,690 (20.0%)
	Middle	4,792 (20.4%)
	High	13,963 (59.6%)
Currently breastfeeding	No	14,547 (62.0%)
	Yes	8,898 (38.0%)
Child alive at interview	Dead	754 (3.2%)
	Alive	22,691 (96.8%)
Birth interval	<24 months	11,599 (49.5%)
	≥24 months	11,846 (50.5%)
Total children	≤5 children	8,634 (36.8%)
	≥6 children	14,811 (63.2%)
Delivery by caesarean section	No	23,384 (99.7%)
	Yes	61 (0.3%)

### Multivariable analysis

Bivariate logistic regression was initially performed for all candidate explanatory variables. Region of residence was examined as a potential determinant of cesarean section delivery; however, it was not significantly associated with the outcome overall (Likelihood Ratio χ^2^ = 19.072, df = 15, *p* = 0.210) and was therefore excluded from the final multivariable logistic regression model. Similarly, educational level showed no overall association with cesarean section delivery (Wald χ^2^ = 1.156, df = 3, *p* = 0.764). Sex of the child (COR = 1.13, 95% CI: 0.68–1.88; *p* = 0.628), child survival status at the time of the interview (COR = 1.02, 95% CI: 0.25–4.18; *p* = 0.978), and preceding birth interval (COR = 1.58, 95% CI: 0.94–2.64; *p* = 0.083) were also not significantly associated with cesarean section delivery. Consequently, these variables were not retained in the final multivariable logistic regression model. Maternal age, place of residence, household wealth, parity, and current breastfeeding status were subsequently entered into the multivariable model.

Compared with women aged ≥ 35 years, those aged 25–34 years had approximately 6.5 times higher odds of cesarean delivery (AOR = 6.48; 95% CI: 2.16–19.46; *p* = 0.001), while women aged < 25 years had approximately 6.3 times higher odds (AOR = 6.29; 95% CI: 2.42–16.35; *p* < 0.001).

Women residing in rural areas had 82.3% lower odds of cesarean delivery than urban residents (AOR = 0.18; 95% CI: 0.09–0.35; *p* < 0.001).

Compared with women from high-income households, those from low-income households had 75.7% lower odds of cesarean delivery (AOR = 0.24; 95% CI: 0.10–0.61; *p* = 0.003). Women from middle-income households also had lower odds of cesarean delivery (AOR = 0.50; 95% CI: 0.20–1.27), although this association was not statistically significant (*p* = 0.145).

Women with six or more children had nearly four times higher odds of cesarean delivery than women with five or fewer children (AOR = 3.96; 95% CI: 2.07–7.57; *p* < 0.001). Women who were currently breastfeeding had lower odds of cesarean delivery (AOR = 0.63; 95% CI: 0.38–1.06), although this association did not reach statistical significance (*p* = 0.079) (Table 2).

**Table 2.** Multivariable logistic regression analysis of factors associated with cesarean section delivery.

Variable	Category	Frequency, n (%)	COR (95% CI)	AOR (95% CI)	*p*-value
Maternal age	≥35 years (Ref)	11,945 (50.9)	1.00	1.00	—
	25–34 years	9,754 (41.6)	17.9 (6.38–50.3)	6.48 (2.16–19.5)	0.001
	<25 years	1,746 (7.4)	10.6 (4.19–26.7)	6.29 (2.42–16.4)	<0.001
Place of residence	Urban (Ref)	10,784 (46.0)	1.00	1.00	—
	Rural	12,661 (54.0)	0.187 (0.097–0.359)	0.177 (0.091–0.346)	<0.001
Wealth index	High-income (Ref)	13,963 (59.6)	1.00	1.00	—
	Middle-income	4,792 (20.4)	0.285 (0.114–0.714)	0.498 (0.195–1.27)	0.145
	Low-income	4,690 (20.0)	0.291 (0.116–0.730)	0.243 (0.097–0.611)	0.003
Parity	≤5 children (Ref)	8,634 (36.8)	1.00	1.00	—
	≥6 children	14,811 (63.2)	6.36 (3.45–11.8)	3.96 (2.07–7.57)	<0.001
Currently breastfeeding	No (Ref)	14,547 (62.0)	1.00	1.00	—
	Yes	8,898 (38.0)	0.553 (0.335–0.915)	0.630 (0.377–1.06)	0.079

## Discussion

This study examined the prevalence and determinants of cesarean section delivery among Somali women using nationally representative data from the 2020 SHDS. The study found that cesarean section delivery was exceptionally rare in Somalia, with substantial socioeconomic and geographic disparities in its utilization. Maternal age, place of residence, household wealth, and parity were associated with cesarean delivery, highlighting disparities in access to emergency obstetric care within the Somali health system.

## Maternal age and cesarean section

In the current study, women aged 25–34 years and those younger than 25 years had higher odds of cesarean delivery than women aged 35 years or older. This finding contrasts with studies from Ethiopia [16], Kenya [17], and Tanzania [17], where advanced maternal age has been associated with higher cesarean section rates.

The observed age pattern may partly reflect the characteristics of the survey sample, as the age distribution was determined by the survey’s sampling frame and inclusion criteria, which required women aged 15–49 years with complete birth histories and complete information on the variables included in the analysis, rather than representing the age distribution of all pregnant Somali women. In addition, Somalia’s unique demographic and healthcare context, where early marriage and early childbearing remain common, may result in a larger proportion of young primigravidas presenting for delivery. Younger women, particularly adolescents and first-time mothers, may also experience greater obstetric complications requiring surgical delivery. Furthermore, younger mothers may have limited reproductive autonomy and reduced ability to advocate for their healthcare needs when making delivery-related decisions, particularly in settings where family members or healthcare providers strongly influence obstetric care. These factors may contribute to the observed age-related differences and warrant further investigation.

## Residence and access to obstetric services

In the current study, women residing in rural areas had substantially lower odds of cesarean delivery than urban residents. This finding is consistent with previous nationally representative evidence from Somalia demonstrating limited access to reproductive health services and substantial socioeconomic and geographic disparities in service utilization, particularly among women living in underserved communities [22]. Similar urban–rural disparities have also been reported across sub-Saharan Africa, where geographic distance, shortages of skilled personnel, limited surgical infrastructure, and financial constraints restrict access to emergency obstetric care [18]. Consequently, women experiencing obstetric emergencies may encounter delays in reaching facilities capable of providing cesarean delivery. These access-related challenges occur within a broader health-system context in Somalia, where recent research has documented shortages of specialized resources and inconsistent access to specialist consultation in emergency care [23].

## Socioeconomic status and cesarean delivery

In the current study, household wealth was associated with cesarean delivery. Women from low-income households had lower odds of cesarean section than women from high-income households. This finding is consistent with studies from Tanzania [24] and Uganda [25]. These similarities may reflect comparable socioeconomic barriers across many low-income settings, including out-of-pocket healthcare expenditures, unequal distribution of specialized obstetric services, transportation costs, and differences in health insurance or financial protection mechanisms. Collectively, these factors may reduce access to emergency obstetric care among economically disadvantaged women.

## Parity and obstetric risk

In the current study, higher parity was associated with cesarean delivery. Women with six or more children had greater odds of cesarean delivery than women with five or fewer children. Similar findings have been reported in other high-fertility settings. Studies from India have shown that grand multiparity is associated with obstetric complications including malpresentation, antepartum hemorrhage, obstructed labor, hypertensive disorders, and postpartum hemorrhage, which may increase the likelihood of cesarean delivery [26]. Likewise, evidence from Saudi Arabia has demonstrated higher rates of placenta previa, placental abruption, and malpresentation among grand multiparous women, resulting in greater operative delivery rates [27].

## Policy implications

The findings have important implications for maternal health policy in Somalia and other conflict-affected, low-resource settings. Expanding comprehensive emergency obstetric and newborn care services beyond major urban centers is essential to reduce geographic inequities in access to life-saving cesarean delivery. Strengthening referral systems, improving ambulance and transport services, and increasing the availability of skilled obstetricians, anesthetists, and surgical infrastructure in underserved areas may reduce delays in accessing emergency obstetric care.

Financial barriers should also be addressed through targeted maternal health financing mechanisms that improve access for low-income households. Furthermore, strengthening person-centered, rights-based family planning programs that respect women’s individual preferences, values, and reproductive goals may help promote healthy birth spacing and reduce preventable obstetric complications while supporting informed reproductive decision-making. Together, these strategies could contribute to improving maternal and neonatal outcomes and reducing longstanding inequities in access to cesarean section services.

## Conclusion

Cesarean section delivery remains exceptionally uncommon in Somalia, highlighting substantial inequities in access to emergency obstetric care. Younger maternal age, higher parity, urban residence, and higher-income household wealth were independently associated with cesarean section delivery, demonstrating persistent socioeconomic and geographic disparities in access to life-saving obstetric services. These findings underscore the need to expand comprehensive emergency obstetric and newborn care, strengthen referral systems, reduce financial and geographic barriers to care, and improve access to person-centered maternal healthcare, particularly for underserved rural and low-income populations. Future research should incorporate clinical information on the indications for cesarean section to distinguish medically indicated from non-medically indicated procedures and further inform maternal health policies and service planning in Somalia.

## Strengths and limitations

This study has several strengths. To our knowledge, it is the first nationally representative study to examine the prevalence and determinants of cesarean section delivery in Somalia using data from the SHDS. The large, nationally representative sample enhances the generalizability of the findings to the target population, while the use of standardized survey procedures and multivariable logistic regression strengthens the reliability and robustness of the analysis.

Nevertheless, several limitations should be considered. First, the cross-sectional design of the SHDS precludes establishing causal relationships between the explanatory variables and cesarean section delivery. Second, the analysis relied on self-reported survey data, which may be subject to recall bias. Third, because the SHDS does not collect detailed clinical information on the indications for cesarean section, it was not possible to distinguish between medically indicated and non-medically indicated (elective) cesarean deliveries. Consequently, although the findings demonstrate a very low cesarean section rate, the study cannot determine the proportion of women who required but did not receive a cesarean section or those who may have undergone the procedure without a clear medical indication. Finally, as this was a secondary analysis of an existing nationally representative dataset, the study population and age distribution were determined by the original SHDS sampling design and eligibility criteria and could not be modified by the investigators.

## Data Availability

This study used secondary data from the 2020 SHDS. The dataset is publicly available through the Somali National Data Archive (SoNADA) and can be accessed from the Somali National Bureau of Statistics data portal. No new data were generated or analyzed for this study.
